# Sex Differences in Inflammation During Venous Remodeling of Arteriovenous Fistulae

**DOI:** 10.3389/fcvm.2021.715114

**Published:** 2021-07-21

**Authors:** Shin Mei Chan, Gabe Weininger, John Langford, Daniel Jane-Wit, Alan Dardik

**Affiliations:** ^1^Vascular Biology and Therapeutics Program, Yale School of Medicine, New Haven, CT, United States; ^2^Department of Surgery, Yale School of Medicine, New Haven, CT, United States; ^3^Division of Cardiovascular Medicine, Department of Internal Medicine, Yale School of Medicine, New Haven, CT, United States; ^4^Department of Immunobiology, Yale School of Medicine, New Haven, CT, United States; ^5^Department of Surgery, Veterans Affairs (VA) Connecticut Healthcare System, West Haven, CT, United States

**Keywords:** vascular inflammation, arteriovenous fistulae, sex differences, estrogens, androgens sex differences in venous inflammation

## Abstract

Vascular disorders frequently have differing clinical presentations among women and men. Sex differences exist in vascular access for hemodialysis; women have reduced rates of arteriovenous fistula (AVF) maturation as well as fistula utilization compared with men. Inflammation is increasingly implicated in both clinical studies and animal models as a potent mechanism driving AVF maturation, especially in vessel dilation and wall thickening, that allows venous remodeling to the fistula environment to support hemodialysis. Sex differences have long been recognized in arterial remodeling and diseases, with men having increased cardiovascular events compared with pre-menopausal women. Many of these arterial diseases are driven by inflammation that is similar to the inflammation during AVF maturation. Improved understanding of sex differences in inflammation during vascular remodeling may suggest sex-specific vascular therapies to improve AVF success.

## Introduction

Although sex differences exist in the epidemiology and clinical presentation of vascular pathologies, patient sex rarely plays a role in guiding medical or surgical management or specific therapeutic treatments. Sex differences in arterial pathologies have frequently been observed in clinical practice, with women presenting with symptoms of coronary artery disease at later ages and with different presentations compared with men; the later age of presentation is frequently after menopause when estrogen levels decrease (1). One explanation is that female hormones provide an anti-inflammatory effect; both estrogen and estrogen receptors exert cardioprotective effects by attenuating inflammatory cytokines, including interleukin-8 (IL-8) and monocyte chemoattractant protein-1 (MCP-1) as well as recruitment of leukocytes (2).

A global rise in end-stage renal disease has resulted in increased placement of autogenous arteriovenous fistulae (AVF), particularly in light of the “Fistula First Breakthrough Initiative” published by the National Kidney Foundation in 2003 (3). This campaign pushed to attain a target of 40% of autologous AVF in the United States by 2006, and then 66% by 2009 (4). Despite these efforts to increase AVF use, up to 60% of AVF fail to mature by 5 months contributing to significant patient burden and healthcare cost (3, 5). There are several predictors of successful AVF adaptation to the fistula environment and use as a successful conduit for hemodialysis, although none are perfect (6). Vein diameter is the most predictive factor in many studies, with larger preoperative vein diameters correlating with higher rates of maturation (7, 8). Other factors, such as diabetes, congestive heart failure, concomitant peripheral arterial disease, and older age may also play a role, as these are negatively correlated with successful AVF creation (5, 8).

Female sex also predicts poor fistula outcomes. Women are less likely to undergo fistula surgery, and when placed, AVF created in women take longer to mature and have higher rates of non-maturation compared with men (3, 9). In elderly patients, at 6 months post-surgery, women are less likely to be successfully dialyzed via their fistula, and at 1 year post-surgery, fistulae are more likely to be completely abandoned in women (10). In women, the time to fistula maturation may also be prolonged compared with men, and women also require more frequent salvage procedures (9, 11). Several hypotheses have been proposed to explain the sex discrepancy in rates of AVF maturation, including smaller mean vessel diameters, greater vascular reactivity following vascular injury, and decreased capacity for venous dilation in women (9). Given the worse clinical outcomes of AVF in women and growing evidence of sex differences in both venous and arterial inflammation, we review the evidence for sex differences in inflammation that occurs during venous remodeling that may contribute to discrepancies in AVF maturation.

## Venous Remodeling

Surgical creation of an AVF results in remodeling of the venous outflow; successful hemodialysis depends on venous remodeling, that is venous dilation and wall thickening, to withstand the high flows required for efficient hemodialysis sessions and puncture with large bore needles 3 times a week (12). Venous remodeling has been studied frequently in the context of vein graft adaption; inflammation regulates vein graft adaptation and sex differences in inflammation may be a mechanism of the reduced vein graft patency among women (13, 14). Analysis of the Project of *Ex Vivo* Vein Graft Engineering via Transfection III (PREVENT III) clinical trial showed that both sex and race predict vein graft patency; black women are more likely to experience loss of vein graft patency and major amputation following bypass surgery (15). However, although AVF maturation and vein graft adaptation both reflect venous remodeling, these processes differ as they take place in different environments and result in different structures (12).

Animal models have shown that venous remodeling during AVF maturation is characterized by increased expression of the venous determinant Ephrin type B receptor 4 (Eph-B4) and the arterial determinant Ephrin-B2, as well as temporal regulation of expression of multiple components of the extracellular matrix (ECM), allowing venous adaptation to the fistula environment without loss of structural integrity (16, 17). Members of the ephrin family mediate cell-cell signaling to promote tissue development and remodeling. In embryogenic tissue, ephrins and their Eph receptors regulate angiogenesis and lymphangiogenesis; in adult endothelial tissues, expression of ephrins are associated with arterial and venous remodeling (16, 18). Expression of both Eph-B4 and Ephrin-B2 show plasticity in adults after surgical manipulation; in vein grafts placed in an arterial environment, Eph-B4 expression is decreased, but expression of arterial markers is not increased (19). However, in AVF, expression of both venous markers such as Eph-B4 and arterial markers such as Ephrin-B2 are increased, resulting in venous remodeling via an Akt1-mediated mechanism (16). During vascular remodeling, ephrins are upregulated by tumor necrosis factor alpha (TNF-α) and regulate nuclear factor -κB (NF-κB), a potential pathway regulating inflammatory processes in endothelial cells (20). In addition Ephrin-B2 promotes leukocyte extravasation and infiltration necessary for vascular remodeling associated with arteriosclerosis (21).

Sex differences involving Eph-B4 and Ephrin-B2 within the vascular system have not been described. However, sex differences have been described in downstream targets. One target of Eph-B4 is Akt, which phosphorylates endothelial NO synthase (eNOS), a critical mediator of venous dilation (22). Phosphorylated eNOS is increased in vein grafts, and absence of eNOS prevents thickening and remodeling of the venous wall (22). In an elegant analysis of a murine AVF model, eNOS mediates dilation of the remodeling vein exposed to the fistula environment; overexpression of eNOS was associated with larger diameter and less neointimal hyperplasia, and eNOS knockout was associated with small diameter and increased neointimal hyperplasia. eNOS overexpression was also associated with smoother blood flow streamlines, less shear stress at the vessel wall, luminal fluid vorticity, and radial wall thinning. These data suggest that eNOS increases NO release from endothelial cells to stimulate smooth muscle cell relaxation (23).

Interestingly, in ovariectomized mice, estrogen induces eNOS to produce and release NO from endothelial cells via the Akt pathway, leading to subsequent arterial dilation (24). In human endothelial cells, there is greater eNOS expression and activation in female-derived cells (25). After menopause there are reduced circulating estrogens, with subsequent reduced arterial NO, that likely contributes to the increased risk for cardiovascular events observed in post-menopausal women compared with pre-menopausal women (26–28). Thus, while sex differences in Eph-B4 and Ephrin-B2 expression have not yet been reported, components of the Eph signal transduction pathway shows sex differences, suggesting that this family of ligands and their receptors may play a large role in the observed differences in venous remodeling.

Transforming growth factor (TGF)-β and TGF-β activated kinase-1 (TAK1) are also important mediators of venous remodeling that promotes wall thickening and dilation by regulating ECM deposition, collagen, fibronectin, and lumen dilation necessary for AVF maturation (29, 30). In endothelial cells, TGF-β can stimulate inflammation and fibrosis via expression of cell adhesion molecules such as intercellular adhesion molecule 1 (ICAM-1) and vascular cell adhesion molecule 1 (VCAM-1), matrix metalloproteinases such as MMP-2, and fibronectins (31). Increased expression of TGF-β during early venous remodeling is likely required for initial maturation of fistulae, and may be a mechanism of compensation to hemodynamic changes, but sustained increased TGF-β and platelet derived growth factor (PDGF) expression likely leads to neointimal hyperplasia that contributes to late AVF failure (32). Several TGF-β polymorphisms alter TGF-β expression and may determine late AVF patency rates in human patients (33). However, sex differences in the TGF-β pathway and downstream effects on the ECM have not been adequately assessed during AVF maturation, despite strong evidence that sex hormones directly interact with the TGF-β superfamily (34). It is possible that sex differences in upstream regulatory pathways, such as in toll-like receptors, may also exist (35).

Since remodeling of the vein during AVF maturation results in expression of both arterial and venous identities in the remodeled venous wall, understanding of sex differences in arterial remodeling may suggest mechanisms relevant to venous remodeling (Table 1). Arterial remodeling involves multiple inflammatory processes. Inflammation and vasoactive peptides promote vessel remodeling by promoting the migration of monocytes and macrophages into the vascular wall, mediated by cell adhesion proteins such as ICAM-1 and VCAM (44, 45). Increased shear stress also promotes vascular remodeling by upregulating TNF-α and NF-κB, as well as activating cellular adhesion molecules to recruit leukocytes (46). Sex differences present during arterial remodeling may be relevant to venous remodeling.

**Table 1:** Observed sex differences in clinical studies and animal models.

**Indication**	**Model**	**Inflammatory marker**	**Men:Women**	**Women:Men**	**References**
AVF	Mice	KLF2, eNOS, VCAM-1	↑ in male mice following AVF creation		(36)
	Mice	TGF-β1, TGFβ-R1, α-smooth muscle actin, fibroblast-specific protein-1, CD68+		↑ in female mice following AVF creation	(37)
AAA	Human	Peripheral blood mononuclear cells (Monocytes, B-cells, T cells)	↑ in men with AAA compared with men without AAA (effect not seen in women)		(38)
	Human	MMP-9		↑, compared with men with AAA	(39)
	Rat	Neutrophils	↑ in elastase-perfused rat		(40)
				↓ in elastase-perfused rat	(41)
	Rat	Macrophages	↑ in elastase-perfused rat		
				↓ in elastase-perfused rats	(41)
	Rat	MMP-13	↑ in elastase-perfused rats		(40)
	Rat	BMP, TNF ligands		↓ in elastase-perfused rats	(41)
	Rat	TGF-β and VEGF		↓ in elastase-perfused rats	(41)
	Mice	JNK1 and downstream proMMP9, proMMP2, active MMP2	↑ in elastase-perfused mice		(42)
Atherosclerosis	Human	CD68+, CD3+, macrophage foam cells		↑ in carotid plaque caps	(43)

↑, *Increased and* ↓, *decreased*.

In arterial remodeling, estradiol administration inhibits monocyte migration in a MCP-1-dependent manner (47). In human endothelial cells treated with lipopolysaccharide (LPS) to induce VCAM-1 expression, estradiol decreases VCAM-1 expression to a greater extent, compared with dexamethasone, by inhibiting NF-kB, activator protein-1 (AP-1), and GATA (48). Estradiol can also influence ICAM-1 expression; treatment of endothelial cells with estradiol leads to a shift from the NF-kB pathway to the c-Jun N-terminal kinase (JNK)/AP-1 pathway (49). Estradiol treatment inhibits TNF-α-dependent VCAM-1 and ICAM-1 expression, as well as inhibition of NF-kB via activation of AMP-activated protein kinase (AMPK) and peroxisome proliferator-activated receptor (PPAR)-α (50). Conversely, administration of androgens to both female and male human endothelial cells shows increased TNF-α signaling and greater expression of inflammatory cytokines, and increased VCAM-1 expression (51). Since sex differences are present in inflammation-driven arterial remodeling, it is likely that similar sex differences exist in venous remodeling.

## Venous Dilation

After an AVF is created, early outward remodeling of the vein is driven by Poiseuille's law, whereby contact with the higher arterial pressure, flow and oxygen content leads to increased venous diameter; this increased diameter, and thus increased volume of the vessel, is necessary for the vein to accommodate increased magnitudes of shear stress and volume flow (12). Preoperative vein diameter is a main predictor of clinical AVF success; in a retrospective review, Lauvao et al. showed that vein diameter was the only independent predictor of fistula maturation (odds ratio = 0.15) (8). Of note, women frequently have smaller diameter vessels compared with men; in a mouse AVF model, outflow veins in male mice were larger than female mice immediately after surgery, and remained larger postoperatively (8, 37, 52).

Outward remodeling is driven by immune and inflammatory processes, particularly CD4+ T cells (53). Rats devoid of mature T cells have decreased lumen sizes following AVF surgery and lower inflammatory cell counts at the fistula site; rescue with euthymic CD4+ T cells leads to increased blood flow through the fistula (54). Sex differences have yet to be assessed in the T cell-mediated venous dilation observed during AVF maturation. However, estrogen can influence Th responses, promote Treg cell populations, and mediate IL-17 release (55). Notably, estrogens can dampen inflammatory responses by modulating Th1 responses to Th2 responses (56). In addition, men have higher Th1:Th2 cytokine ratios (57). Future work is necessary to identify sex-associated differences in T cell-mediated outward remodeling in AVF.

MMP may also play sex-dependent roles in inflammatory venous remodeling. MMP are a family of zinc-dependent endopeptidases that degrade collagen and elastin in the ECM and have been implicated in dilatory venous and arterial diseases, including chronic venous insufficiency and aneurysmal disease (58–60). MMP are secreted by various cell types, particularly inflammatory cells (i.e., lymphocytes, macrophages, neutrophils), endothelial cells, and vascular smooth muscle cells (58). In chronic venous disease, multiple MMP have been implicated (58). During AVF maturation, serum levels of MMP-2, MMP-9, and metalloproteinase tissue inhibitors (TIMP) are associated with successful creation of AVF; higher MMP-2/TIMP-2 and MMP-9/TIMP-2 ratios were associated with better prognosis (61).

Notably, the relaxin family of hormones upregulates MMP-2 and MMP-9 expression to contribute to vessel remodeling (62, 63), suggesting that relaxin-relaxin receptor signaling might be a significant contributor to the sex differences present during AVF maturation as relaxin and its downstream molecules differ between sexes (64–68). Knockout of the relaxin receptor resulted in decreased outward remodeling in a murine model of AVF failure, accompanied by increased elastin content, reduced elastase activity, increased CD45+ leukocytes, and increased MCP-1 expression (69). Chronic administration of recombinant relaxin reduces arterial load by decreasing systemic vascular resistance, reduces pulsatile arterial load by increasing compliance, and increases cardiac output in female mice (65); interestingly, there were no sex differences, despite relaxin being considered specific to female physiology (64, 66). Elastins may also be involved in the sex differences of vessel remodeling, as estrogen may decrease MMP-9 production, thus decreasing elastin degradation (70). Elastin haplodeficient mice show increased outward remodeling, without increased intimal hyperplasia, resulting in larger diameter venous outflow tracts (71). Although the data showing elastin regulates venous remodeling is strong in animal models, human clinical trials with recombinant elastase did not show clear improvements in AVF outcomes (72, 73).

**Figure 1:**
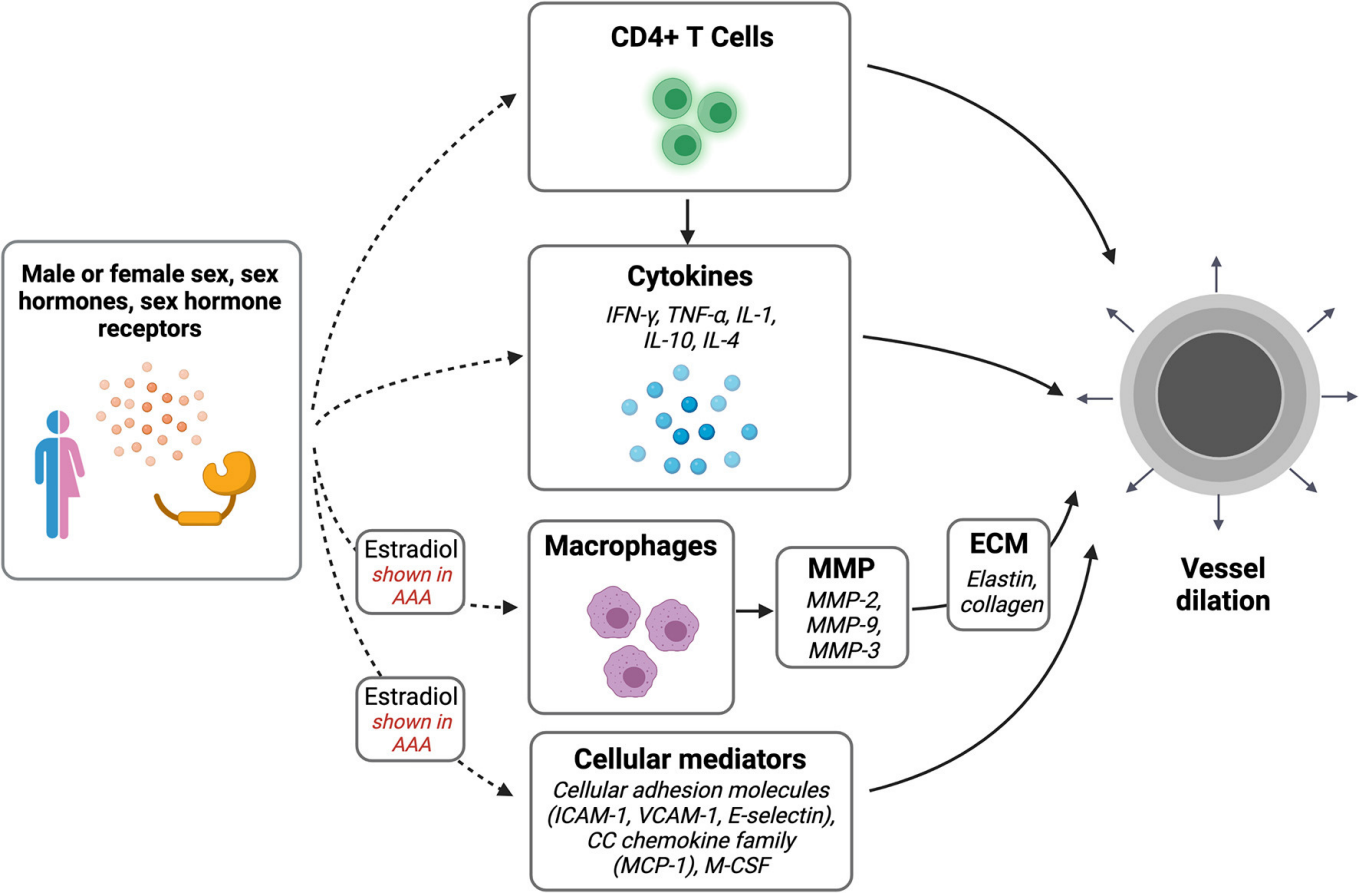
Sex differences in vascular dilation. Potential pathways of interest in vessel wall dilation that may be affected by sex, sex hormones, and sex hormone receptors. Created with BioRender.com.

The inflammatory process of venous outward remodeling may be similar to remodeling that occurs during the formation of arterial aneurysms (Figure 1). Sex differences in arterial aneurysms have been extensively described. Both thoracic aortic aneurysms (TAA) and abdominal aortic aneurysm (AAA) occur less commonly in women than in men (74). Women, however, have worse prognosis; in women, AAA and TAA grow at faster rates, rupture at smaller diameters, and are associated with higher mortality (75–81). Aortic stiffness predicts TAA growth in women but not men, further suggesting a potential sex difference in the pathophysiology (81). In addition, women develop AAA at older ages than men (82), which has been suggested to be associated with menopausal status and decreasing estrogen levels (39).

Similar to venous remodeling, AAA are driven by T cell-mediated inflammatory processes (Table 2); both CD4^+^ and CD8^+^ T-cells are highly activated in the aortic wall, and perivascular T cell counts correlate with aneurysm progression (89). Th2 cytokines, particularly IL-1, IL-4, and IL-10, simultaneously activates MMP, which promotes the outward remodeling of the aneurysmal wall (90–92). Estradiol attenuates inflammatory cytokines and chemokines necessary for MMP-2 and MMP-9 release and monocyte infiltration, which protect against AAA; this may be mediated through the JNK pathway (42, 70). Similarly, following aortic elastase perfusion in male rats there are increased numbers of infiltrating macrophages compared with female rats, and treatment with estrogen in male rats leads to smaller aneurysms accompanied by decreased macrophage infiltration (41, 70). Human aortic smooth muscle tissue incubated in testosterone showed increased MMP-3 expression, whereas incubation with estrogen and progesterone reduced collagen deposition and increased elastin deposition (83). In rats, MMP-13 expression is increased in male rats perfused with elastase compared with female rats (40). In mice, increased estrogen receptor-α (ER-α) in female mice was inversely correlated with MMP activity and aneurysm formation (84). Estrogen administration decreases MMP-2 and−9 expression, adhesion molecule expression and macrophage stimulators such as ICAM-1, VCAM-2, E-selectin, MCP-1, and macrophage colony-stimulating factor (M-CSF) (38, 70, 85). Since estrogen plays a protective mechanism in AAA formation via the inflammatory MMP pathway, further research is needed to understand the contribution of this mechanism to fistula maturation.

**Table 2:** Differences in vascular inflammation due to sex hormones in clinical studies and animal models.

**Indication**	**Model**	**Inflammatory marker**	**Androgen-related**	**Estrogen-Related**	**References**
AAA	Human	MMP-3	↑		(83)
	Mice	MMP-2, MMP-9		↓ (inversely correlated with estrogen receptor α)	(84)
				Ovariectomy resulted in higher levels of MMP-2 and MMP-9	(38)
				↓ in male rats given estradiol	(70)
	Mice	ICAM-1, VCAM-2, E-selectin, MCP-1, M-CSF		↓	(85)
	Mice	IL-1α mediated inflammatory response (MCP-1, MMP-2, MMP-9), TGF-β	↑ (knock-out of AR leads to ↓)		(86)
Atherosclerosis	Human	Monocyte migration		↓, in a MCP-1 dependent manner	(47)
	Human	VCAM-1	↑		(51)
				↓ via NF-kB, AP-1, GATA inhibition	(48)
				↓ via TNF- α, NF-kB inhibition	(50)
	Human	ICAM-1	No significant change		(51)
				↓ via TNF- α, NF-kB inhibition	(50)
				Causes shift in pathway (NF-kB JNK/AP-1)	(49)
	Human	E-selectin	No significant change		(51)
	Rats	Adhesion molecules (P-selectin, VCAM-1, ICAM-1), monocyte chemoattractant (CINC-2β, MCP-1), proinflammatory mediators (IL-1, IL-6)		↓	(87)
	Mice	SOCS3		↑	(88)
	Mice	JAK/STAT		↓	(88)

↑, *Increased and* ↓, *decreased*.

## Venous Wall Thickening

Vascular remodeling is composed of both changes in vessel diameter as well-changes in wall thickness. Excessive venous neointimal thickening leads to late AVF failure, observed more commonly in women than men (9). Venous neointimal thickening also depends on Eph function; in mouse models, decreased Eph-B4 signaling is associated with increased venous wall thickening (16, 93). Eph-B4 also regulates wall thickening in human veins, at least *in vitro* (94–96).

Interestingly, recent data using a mouse AVF model has shown that Kruppel-like factor 2 (KLF2), eNOS, and VCAM-1 increase in male but not female mice, despite being similar at baseline prior to AVF creation; in addition, female mice showed reduced laminar shear stress that was followed by reduced AVF patency at 42 days (36). Transcriptome RNA sequencing has also showed that inflammatory pathways are differentially upregulated in male mice following AVF creation; expression of proinflammatory cytokine IL-17 was less in females, but fibrotic markers TGF-β1 and TGF-β receptor 1 were increased, with correlation to negative vascular remodeling and increased medial fibrosis (36, 37). HIF-1α and heme oxygenase-1/2 are also inflammatory mediators of neointimal hyperplasia that forms during AVF maturation and that may differ between sexes (97–99). Although partial HIF-1α deficiency regulates differential changes in cardiac gene expression between female and male mice (100), and heme oxygenase expression differs in the setting of trauma and hemorrhage (101), sex differences in these mediators have not been studied in the setting of venous remodeling.

Macrophages and T cells contribute significantly to venous neointimal hyperplasia and may further contribute to sex differences observed during AVF maturation (53). Following angioplasty of fistulae, female mice had increased neointimal area-to-media ratio, accompanied by increased numbers of CD68+ cells, suggesting sex differences in macrophages during venous remodeling (102). M2-type macrophages are important for vascular wall thickening by secreting IL-10 that is necessary for wall thickening during AVF maturation (53, 103). Rapamycin, an immunosuppressant, decreases AVF wall thickness, ECM deposition, and smooth muscle cell proliferation via suppression of both M1-type and M2-type macrophages (104). The fractalkine receptor 1 (CX3CR1) reduces venous stenosis in AVF by decreasing proinflammatory signaling, including TNF- α, IL-1β, MCP-1, and NF-kB (105). In a murine AVF model, liposomal prednisolone inhibits venous inflammation and improves outward remodeling; inflammatory cytokine release from M1-type macrophages was reduced, suggesting conversion of macrophages into an anti-inflammatory profile (106). However, no significant effects on neointimal hyperplasia were observed, similar to the lack of efficacy observed in the human Liposomal Prednisolone to Improve Hemodialysis Fistula Maturation (LIPMAT) clinical trial (106, 107). These data suggest that the role of inflammatory signaling during AVF maturation is complex, and additional studies that separately examine the role of inflammation during early maturation and later patency are warranted.

**Figure 2:**
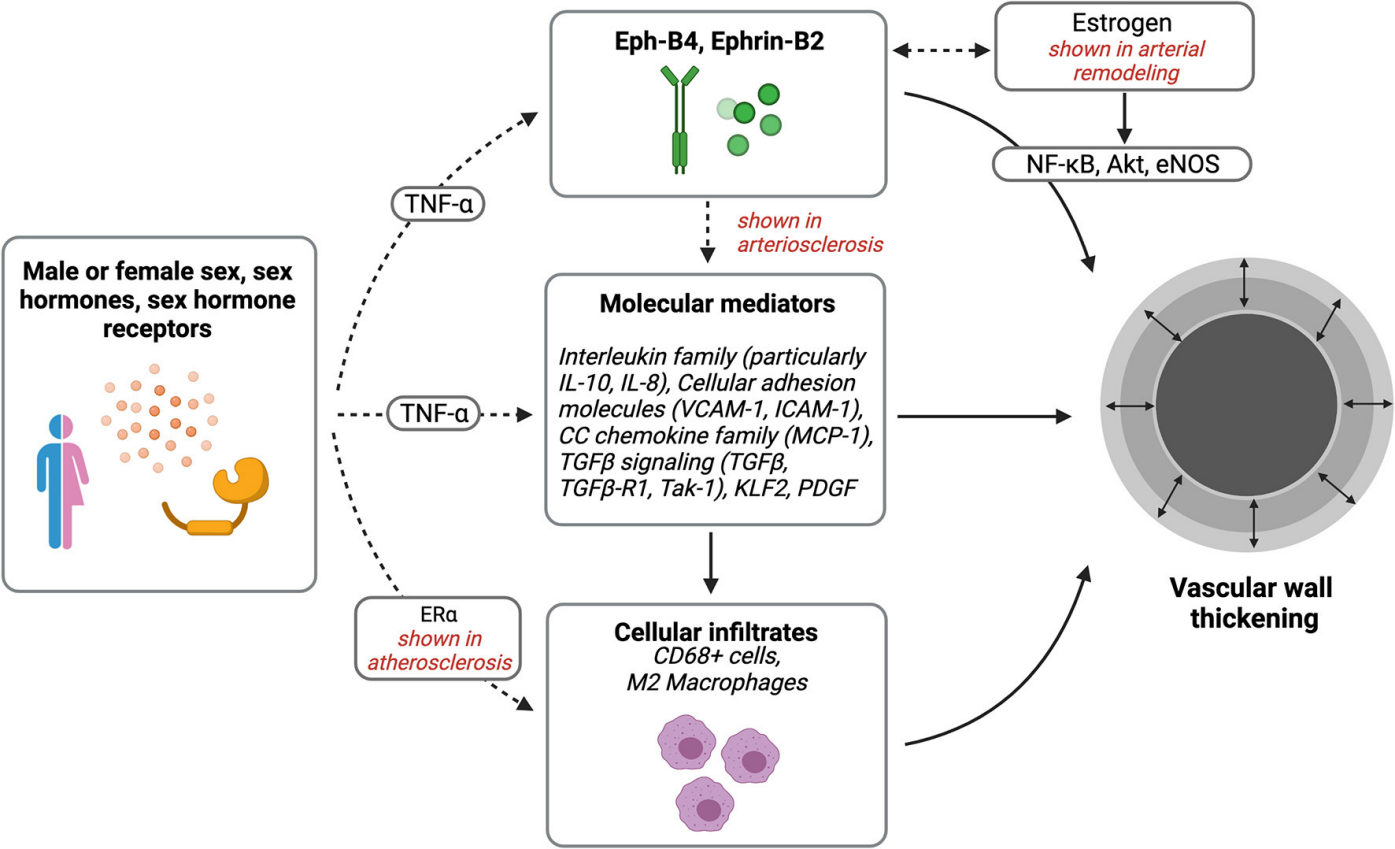
Sex differences in vascular wall thickening. Potential pathways of interest in vessel wall thickening that may be affected by sex, sex hormones, and sex hormone receptors. Created with BioRender.com.

In both atherosclerosis and arterial neointimal hyperplasia, macrophages have been implicated in sex differences (Figure 2) (108). Notably, men have more frequent atherosclerotic-related events and at younger ages compared with women, but cardiovascular diseases increase in women after menopause, suggesting significant sex hormone-dependent factors (109, 110). Sex differences are also present in the content of inflammatory cells in atherosclerotic lesions; for example, carotid plaque caps show more CD68+ and CD3+ inflammatory cells, including macrophage foam cells, in women compared with men (43). Macrophages express sex hormone receptors; higher levels of ER-α in premenopausal women are associated with a lower incidence of atherosclerosis (108). In addition, estradiol can attenuate macrophage foam cell formation within atherosclerotic plaques, via JAK/STAT/SOC3 pathway (88). Interestingly, administration of androgens to human male-derived macrophages showed increased expression of inflammatory genes associated with atherosclerosis, but not in female-derived macrophages, further suggesting differential susceptibilities to atherosclerosis based on sex (111). Thus, similar to sex differences observed in arterial neointimal hyperplasia, macrophages may contribute to sex-related differences in late AVF failure.

Vascular smooth muscle cells also play a dynamic role during AVF maturation. Differentiated, mature smooth muscle cells primarily contribute to medial wall thickening and dedifferentiated smooth muscle cells contribute to neointimal hyperplasia (112, 113). Sex differences in smooth muscle cell function have not been directly studied during AVF remodeling; however, it is reasonable that these differences exist, as studies in arterial neointimal hyperplasia have shown that smooth muscle cells function in a sex-dependent manner. In vein grafts implanted into arterial environments, Eph-B4 is found in both endothelial cells and smooth muscle cells, although Eph-B4 is preferentially distributed in endothelial cells (93). In cell culture, female-derived smooth muscle cells show more hypertrophic changes, whereas male-derived smooth muscle cells show more hyperplastic changes; additionally, female-derived cells were more adhesive, suggesting slower proliferation (114). In female rats, increased ER in smooth muscle cells was associated with reduced arterial contraction (115). Mineralocorticoid receptors in smooth muscle cells mediate later onset of aortic stiffening and vascular fibrosis in female mice compared with male mice (116). These studies suggest that sex differences in smooth muscle cell function may also be a mechanism of the sex differences observed during AVF maturation. However, the failed PREVENT trials suggest that inhibition of smooth muscle cell proliferation during vein graft adaptation is unlikely to be a clinically successful strategy, and thus inhibition of smooth muscle cell function during AVF maturation would require another approach to optimize venous remodeling (117, 118).

## Conclusion

AVF maturation and utilization remains poor, particularly in women. There is growing understanding of the inflammatory mechanisms that drive successful AVF maturation and may differ between men and women. Successful AVF creation depends on venous dilation and wall thickening, both of which involve inflammatory mechanisms. Outward remodeling is dependent on CD4+ T cells, and venous hyperplasia is dependent on distinct subsets of macrophages. In arterial aneurysms and atherosclerosis, where sex differences have long been recognized, there is a larger body of evidence supporting sex differences in inflammatory vessel remodeling. Mechanisms of arterial remodeling may help guide understanding of venous remodeling and may lead to improved clinical outcomes for women needing AVF.

## Author Contributions

SC and AD contributed to the conception and design of the study. SC, GW, JL, and AD performed the literature search, analyzed the data, synthesized the literature, and were responsible for writing the manuscript. DJ-W and AD provided critical expertise and expert revision. All authors contributed to manuscript revision, read, and approved the submitted version.

## Conflict of Interest

The authors declare that the research was conducted in the absence of any commercial or financial relationships that could be construed as a potential conflict of interest.

## Abbreviations

AAA: Abdominal aortic aneurysm
AMPK: 5′ adenosine monophosphate-activated protein kinase
AP-1: Activator protein-1
AVF: Arteriovenous fistula
BMP: Bone morphogenic protein
CC: Cysteine-cysteine
CD: Cluster of differentiation
CINC: Cytokine-induced neutrophil chemoattractant
CX3CR1: Cysteine-X3-cysteine motif chemokine receptor 1
ECM: Extracellular matrix
eNOS: Endothelial nitric oxide synthase
Eph-B4: Ephrin type B receptor 4
ER-α: Estrogen receptor-α
ICAM-1: Intercellular adhesion molecule 1
IL: Interleukin
JAK: Janus kinase
JNK: c-Jun N-terminal kinase
KLF2: Kruppel-like factor 2
LPS: Lipopolysaccharide
M-CSF: Macrophage colony-stimulating factor
MCP-1: Monocyte chemoattractant protein-1
MMP: Matrix metalloproteinase
NF-κB: Nuclear factor-κB
PDGF: Platelet derived growth factor
PPAR: Peroxisome proliferator-activated receptor
SOC3: Suppressor of cytokine signaling
STAT: Signal transducer and activator of transcription
TAA: Thoracic abdominal aneurysm
TAK1: TGF-β activated kinase-1
TGF-β: Transforming growth factor-β
TIMP: Tissue inhibitor of metalloproteinases
TNF-α: Tumor necrosis factor-α
VCAM-1: Vascular cell adhesion molecule-1.
